# The Importance of Community and Patient Involvement in the Design of Physical Activity Programs

**DOI:** 10.1177/2150132720957444

**Published:** 2020-09-10

**Authors:** Mohammad S. Razai, Pippa Oakeshott

**Affiliations:** 1 St George’s University of London, London, UK

In a fascinating article, Harrison and colleagues examined the perceptions, beliefs, and opinions of older adults about physical activity and exercise.^1^ We agree that qualitative assessment by potential users is crucial in the development and evaluation of any physical activity program. Our experience of engaging with members of Action on Preeclampsia (APEC) and our local King’s College London Preeclampsia PPI (patient and public involvement) group in planning a trial of a postnatal physical activity program demonstrate the issues raised by Harrison and colleagues regarding barriers and motivators for physical activity. We used a free mobile health app to encourage brisk walking in postnatal women, focusing on those who have had a hypertensive disorder of pregnancy. Like the 71% of Harrison’s participants with hypertension,^1^ these women are at increased long-term risk of future hypertension and cardiovascular death,^2^ but increasing physical activity with an app should reduce these risks.^3^

We systematically went through several stages to explore the functionality and usability of the mobile health app, discussing our proposal with members of APEC, then asking members of our Preeclampsia PPI group to try the app, and finally conducting a service evaluation pilot in ethnically diverse postnatal women at 2 inner city general practices. At follow up after ≤3 months we found that 18 out of 24 (75%) postnatal women who were given a leaflet promoting the app, had used it (14) or another (4) exercise app, and all reported that they had increased their brisk walking. Comments included: “It helps me keep track of weeks when I am more or less active.” “I use it and would recommend it.” “App is good. I used it for two weeks and walk faster.” “Am doing more brisk walking.” “I need to keep healthy to look after my baby.” “I don’t like exercise but I could walk faster.”

By going through this systematic evaluation at an early stage, service providers and researchers could find out valuable information from patients about their preferences, beliefs and barriers to physical activity. Similarly, the functionality and usability of any program such as a mobile app could be refined based on feedback from users. We agree with Harrison and colleagues that involving patients and service users is absolutely critical to the success of any physical activity program and can help guide policy.^1^ Furthermore, we must ensure that researchers follow established protocols for the evaluation of mobile health apps^4^ and other physical activity interventions.
